# CXCR2 Is Essential for Radiation-Induced Intestinal Injury by Initiating Neutrophil Infiltration

**DOI:** 10.1155/2022/7966089

**Published:** 2022-07-16

**Authors:** Jing Hu, Qian Ji, Fei Chen, Xiaoqin Gong, Chuansheng Chen, Kaijun Zhang, Ye Hua, Jinlei Wang, Rongkun Li, Xu Wang, Chunhua Dai, Ye Tian

**Affiliations:** ^1^Department of Radiation Oncology, The Second Affiliated Hospital of Soochow University, Suzhou 215000, China; ^2^Department of Radiation Oncology, Affiliated Hospital of Jiangsu University, Zhenjiang 212000, China

## Abstract

Neutrophils, known as an important part of the immune system, are the most abundant leukocyte population in peripheral blood, but excessive recruitment will lead to tissue/organ injury. RNA sequencing showed that ionizing radiation significantly increased the expression of characteristic genes of neutrophils in intestinal tissues compared with liver and lung tissues. By clearing neutrophils with an anti-Ly6G antibody, we found that neutrophil infiltration is critical for irradiation-induced intestinal injury. CXCR2 is a G-protein-coupled receptor that mediates the migration of neutrophils by combining with its ligands. Compared with observations in liver and lung tissues, we found that CXCR2 and its ligands, including CXCL1, CXCL2, CXCL3, and CXCL5, were all significantly upregulated in irradiated intestinal tissues. Further studies showed that SB225002, an inhibitor of CXCR2, could effectively inhibit the chemotaxis of neutrophils and tissue damage mediated by the CXCL-CXCR2 signalling pathway.

## 1. Introduction

Radiation enteritis is one of the most common complications of the intestinal tract caused by radiotherapy for malignant tumours in the abdomen and pelvic cavity [1]. The main clinical manifestations are abdominal pain, diarrhoea, malabsorption, and even intestinal obstruction, bleeding, and intestinal perforation, which may occur in severe cases [2, 3]. Radiation enteritis can seriously reduce the quality of life of patients, lead to treatment interruption, and even affect clinical efficacy [4, 5]. The occurrence and development mechanism of radiation enteritis is complex and mainly involves intestinal epithelial cell injury, intestinal vascular endothelial cell injury, intestinal flora imbalance, and inflammatory factor imbalance [6–9]. Moreover, impairment of intestinal barrier function can lead to extensive bacteria and endotoxin invasion, resulting in sepsis, endotoxaemia, and other systemic inflammatory response syndromes, which can be life-threatening [10, 11]. No standard treatment is available for irradiation-induced intestinal injury, and the main purpose of clinical treatment strategies is to alleviate the symptoms of intestinal reactions in patients after irradiation [12–14]. Neutrophils, the most abundant cells in the innate immune system, are typically the first leukocytes to be recruited to inflammatory sites to defend against infection or inflammation [15]. In recent years, accumulating evidence has shown that neutrophil infiltration plays an important role in intestinal inflammation and tumour progression, and excessive recruitment of neutrophils can lead to tissue/organ injury [16–19]. Inhibition of neutrophil migration may provide a new therapeutic strategy for radiation enteritis.

CXC motif chemokine receptor 2 (CXCR2), a G-protein-coupled receptor, is widely expressed in neutrophils, lymphocytes, and other types of nonhaematopoietic cells. It mediates the migration of neutrophils to inflammatory sites by combining with its ligands. It is also involved in the progression of angiogenesis, inflammation, wound healing, and tumour formation [20–22]. Studies have shown that the CXC ligand- (CXCL-) CXCR2 signalling pathway contributes to neutrophil chemotaxis in response to tissue injury and infections [23, 24]. Blocking CXCR2 can alleviate acute pancreatitis, haemorrhagic cystitis caused by cyclophosphamide, and acute experimental colitis in mice [25–28]. However, whether CXCR2 inhibitors have a protective effect on irradiation-induced intestinal injury has not been reported.

In this study, RNA sequencing was performed to explore the potential mechanism of acute radiation enteritis. Through high-throughput data analysis, we found that the characteristic genes of neutrophils were significantly upregulated in intestinal tissue after irradiation. In addition, CXCR2 and its ligands were significantly increased. Therefore, we hypothesize that the CXCL-CXCR2 signalling pathway is essential for irradiation-induced intestinal injury by initiating neutrophil infiltration. Targeting CXCR2 and its ligands may provide a new strategy for the treatment of radiation enteritis.

## 2. Materials and Methods

### 2.1. Animal Models

All animal experiments were carried out in compliance with the National Institutes of Health Guide for The Care and Use of Laboratory Animals and approved by the experimental animal ethical committee of Jiangsu University. We used 6- to 8-week-old C57BL/6 mice weighing approximately 25 g with free access to food and water. Mice were randomly divided into four groups, including the control group, the irradiation alone group, the irradiation+anti-Ly6G antibody group, and the irradiation+SB225002 group. Mice in the anti-Ly6G antibody group were injected intraperitoneally with anti-Ly6G antibody (Biolegend, catalogue no. 127649) at a dose of 50 *μ*g/mouse/day three times, 24 hours, 1 hour before irradiation, and 24 hours after irradiation. Mice in the SB225002 group were injected intraperitoneally with SB225002 (MedChemExpress, catalogue no. 182498-32-4) at a dose of 2 mg/kg/d at the same time as the anti-Ly6G antibody injection. The negative control group and the irradiation alone group were injected intraperitoneally with normal saline at the same time. Mice were sacrificed 48 hours after irradiation, and their liver, lung, and intestine tissues were removed, fixed with 10% formalin, and preserved with liquid nitrogen.

### 2.2. IR

After anaesthetization with ether, the mice were supine and fixed on a foam board for whole-body irradiation. A Varian 23EX linear accelerator (USA) was used. The irradiation dose was 14 Gy, and the dose rate was 400 Mu/min.

### 2.3. RNA Sequencing

According to the manufacturer's instructions, total RNA was isolated from liver, lung, and intestine tissues of mice after irradiation using TRIzol reagent and sent to Novogene Co., Ltd. for clustering and sequencing to obtain relevant RNA sequencing data.

### 2.4. Haematoxylin-Eosin (H&E) Staining

The liver, lung, and intestine tissues of mice were fixed with 10% formalin and sectioned at a thickness of 5 *μ*m after paraffin embedding. The slices were dewaxed, hydrated, and stained with haematoxylin and eosin. Then, the slices were dehydrated and transparentized, and the tissues were observed under a microscope after the slices were sealed. Pathologists assessed the histological severity of irradiation-induced intestinal injury by the degree of epithelial structure maintenance, crypt damage, vasodilation, and inflammatory cell infiltration (0 = no damage, 1 = mild, 2 = moderate, and 3 = high).

### 2.5. Myeloperoxidase (MPO) Activity

The liver, lung, and intestine tissues of mice were immediately frozen in liquid nitrogen after extraction. Then, the tissues were homogenized and centrifuged, and the supernatant was collected. Finally, MPO activity in the tissues was determined using an MPO activity detection kit according to the manufacturer's instructions.

### 2.6. PCR

Total RNA was extracted from tissues using TRIzol reagent (Takara), and RNA was reverse transcribed to cDNA using PrimeScript RT–PCR Kit (Takara) according to the manufacturer's instructions. Real-time PCR was performed using SYBR Premix Ex Taq (Roche) to analyse gene expression. Relative mRNA expression was calculated using the 2^−*ΔΔ*Ct^ method and normalized to the mRNA level of *β*-actin.

### 2.7. Immunohistochemistry (IHC)

The liver, lung, and intestine tissues of mice were fixed with 10% formalin and sectioned at a thickness of 5 *μ*m after paraffin embedding. Then, the slices were dewaxed, hydrated, and placed into hydrogen peroxide solution to block endogenous peroxidase after antigen repair. Later, the slices were stained after BSA sealing and redyed with haematoxylin. Finally, the slices were dehydrated, transparentized, and sealed.

### 2.8. Statistical Analysis

Data are expressed as the mean ± standard deviation (mean ± SD) and were analysed using GraphPad Prism 8 software. All experiments were repeated at least three times. Student's *t* test was used for comparisons between the two groups. One-way analysis of variance (ANOVA) and Tukey's test were used for comparisons between multiple groups. *p* < 0.05 was considered statistically significant.

## 3. Results

### 3.1. Transcriptome Changes in the Liver, Lung, and Intestine of Mice after Irradiation

**Figure 1:**
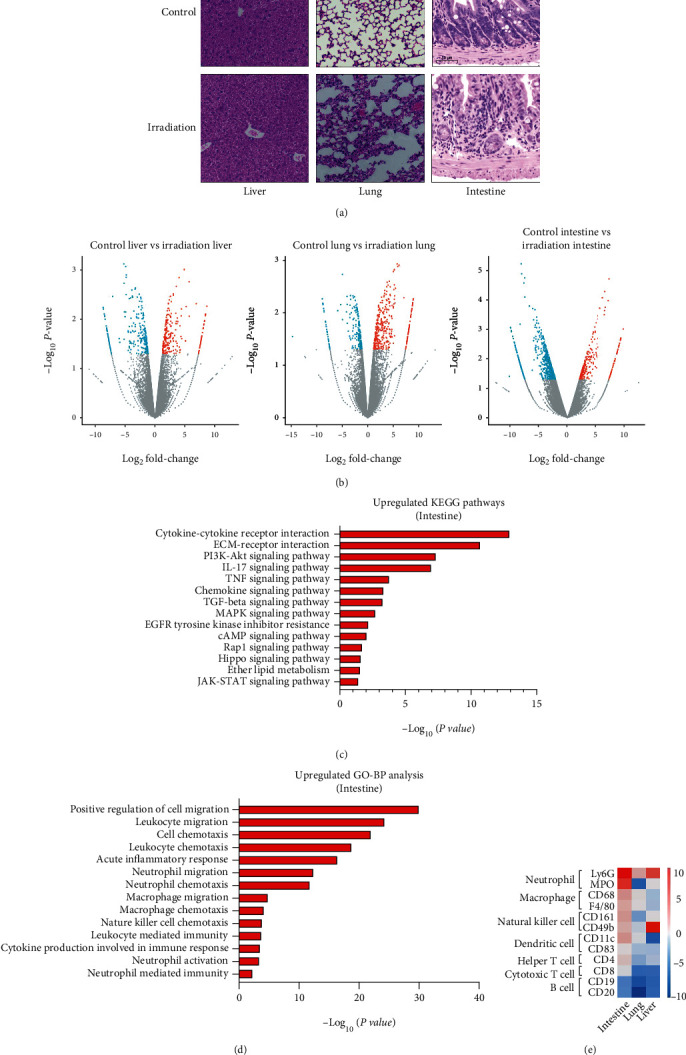
Transcriptome changes in the liver, lung, and intestine of mice after irradiation. (a) Representative images of the H&E staining in the liver, lung, and intestine from control and irradiated mice at day 3. The scale bar is 50 *μ*m. (b) RNA transcription profile changes in liver, lung, and intestine tissues of mice in the control group and the irradiation group. Blue dots represent downregulated gene expression, and red dots represent upregulated gene expression. (c) KEGG pathway enrichment analysis of differentially expressed genes in the intestinal tissue of mice. (d) GO biological process analysis of differentially expressed genes in the intestinal tissue of mice. (e) Heatmap of characteristic genes of different immune cells through RNA sequencing in the liver, lung, and intestine of mice.

Morphological changes in the liver, lung, and intestine of mice after irradiation were observed by H&E staining. Compared with that in the control group, the damage to liver, lung, and intestinal tissues was more obvious in mice after irradiation (see Figure 1(a)).

To explore the potential mechanism of acute irradiation-induced tissue injury, comparative analysis of transcriptome changes was performed between nonirradiated mice and irradiated mice. The results of differential analysis showed that 268 genes were upregulated and 364 were downregulated in liver tissue. In lung tissue, 218 genes were upregulated, and 364 were downregulated. In intestinal tissue, 704 genes were upregulated, and 278 were downregulated (see Figure 1(b)). To further confirm the changes in relevant signalling pathways after irradiation, KEGG signalling pathway analysis was performed, and we observed significant enrichment of inflammation-related signalling pathways in intestinal tissue. In recent years, many studies have found that inflammatory factors and their related signalling pathways play a key role in the development of radiation enteritis. Radwan and Karam [29] found that TNF-*α*, NF-*κ*B, and IL-1*β* were significantly increased, and the expression levels of PI3K, Akt, and mTOR were significantly upregulated after irradiation. Resveratrol reduced intestinal inflammation after irradiation by inhibiting the PI3K/Akt/mTOR pathway and thus inhibiting the release of proinflammatory cytokines. Sha et al. [30] found that TNF-*α*, IL-1, IL-6, and NF-*κ*B were upregulated and PPAR-*γ* was downregulated after irradiation, while rheinic acid alleviated acute radiation enteritis by activating PPAR-*γ* and inhibiting the NF-*κ*B and p38 MAPK signalling pathways. In this study, pathways including the PI3K-Akt signalling pathway, TNF signalling pathway, and MAPK signalling pathway were also enriched in irradiated intestinal tissues. In addition, this study found that cytokine–cytokine receptor interactions, the IL-17 signalling pathway, and the chemokine signalling pathway were significantly enriched (see Figure 1(c)). Additionally, Gene Ontology (GO) biological process analysis showed that inflammatory immune processes were enriched in irradiated intestinal tissue, including leukocyte migration/chemotaxis, acute inflammatory reaction, neutrophil migration/chemotaxis, leukocyte-mediated immunity, and production of cytokines involved in the immune response (see Figure 1(d)). By comparing the characteristic genes of different immune cells, we found that immune cells, including neutrophils, macrophages, natural killer cells, dendritic cells, and helper T cells, were upregulated in intestinal tissue, whereas cytotoxic T cells and B lymphocytes were downregulated. Among the characteristic immunocyte-associated genes, we found that neutrophil-related genes were the most significantly upregulated (see Figure 1(e)).

### 3.2. Excessive Infiltration of Neutrophils Promotes Intestinal Injury after Irradiation

**Figure 2:**
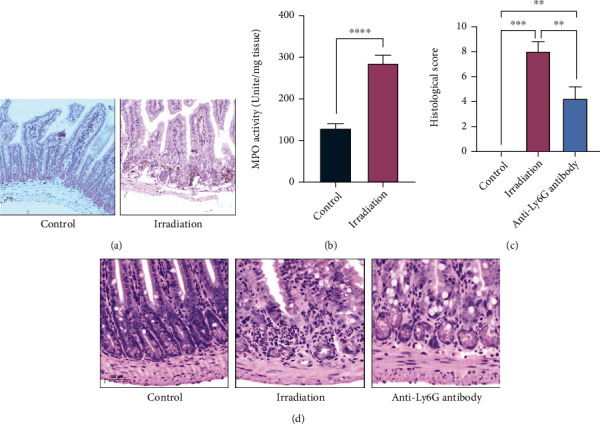
Excessive infiltration of neutrophils promotes intestinal injury after irradiation. (a) Representative images of Ly6G expression in the intestinal tissue of mice in the control group and the irradiation group by IHC staining at day 3. The scale bar is 50 *μ*m. (b) MPO activity of intestinal tissues of mice in the control group and the irradiation group (*n* = 4 per group). (c) Histological scores of intestinal tissues of mice in the control group, the irradiation group, and the irradiation+anti-Ly6G antibody group after H&E staining (*n* = 4 per group). (d) Representative images of the H&E staining of the intestine from the control group, the irradiation alone group, and the irradiation+anti-Ly6G antibody group at day 3. The scale bar is 50 *μ*m. ^∗^*p* < 0.05, ^∗∗^*p* < 0.01, ^∗∗∗^*p* < 0.001, and ^∗∗∗∗^*p* < 0.0001.

Neutrophils, which are known as the “front line” of host defence, are the most abundant immune effector cells in the innate immune system. However, excessive recruitment of neutrophils may release harmful inflammatory mediators, such as cytokines, proteases, and reactive oxygen species, resulting in tissue/organ damage [31]. The results of RNA sequencing showed that the characteristic genes of neutrophils were most significantly upregulated in intestinal tissue after irradiation compared with lung and liver tissue. Compared with those in the control group, the H&E staining results showed that intestinal lesions were aggravated in the irradiation group, including intestinal crypt structural destruction, intestinal mucosa hyperaemia and oedema, and a significant increase in neutrophil infiltration (see Figure 1(a)). The expression of Ly6G (a neutrophil marker) in the intestinal tissues of mice was further detected by immunohistochemistry, and the protein level of Ly6G was found to be significantly increased in the irradiation group (see Figure 2(a)). MPO activity is an effective parameter to evaluate neutrophil infiltration. The results showed that the MPO activity in intestinal tissues of mice in the irradiation group was significantly higher than that in the control group (see Figure 2(b)), suggesting that neutrophil infiltration might have a potential role in irradiation-induced intestinal injury.

Considering the upregulated expression of the characteristic genes of neutrophils, we believe that neutrophil infiltration is more obvious in irradiated intestinal tissue, which may be one of the reasons for irradiation-induced intestinal injury. To determine whether neutrophil infiltration can promote irradiation-induced intestinal injury in mice, we used an anti-Ly6G antibody to deplete neutrophils in mice. H&E staining showed that neutrophil infiltration and intestinal injury were significantly alleviated in mice treated with anti-Ly6G antibody compared with the irradiation alone group (see Figures 2(c) and 2(d)), indicating that neutrophils are essential for radiation enteritis.

### 3.3. The Expression of CXC Chemokine Receptor 2 and Its Ligands Is Increased after Irradiation

**Figure 3:**
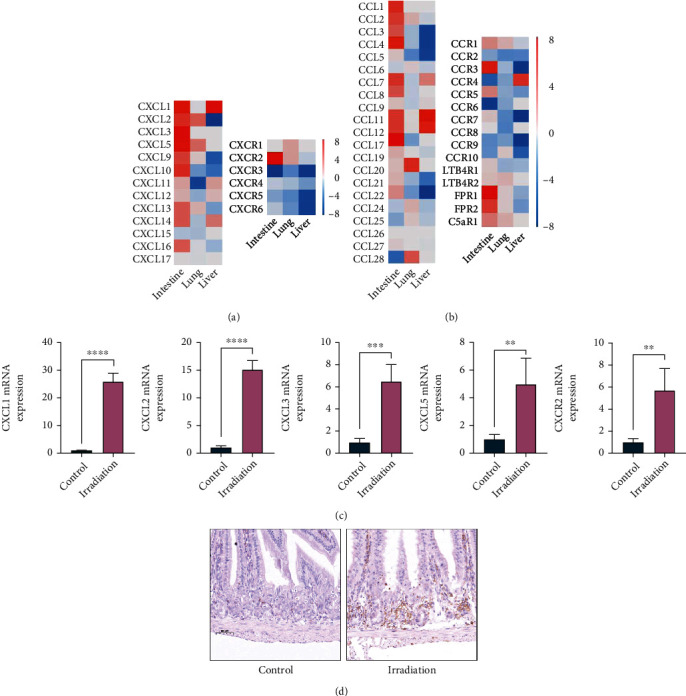
The expression of CXC chemokine receptor 2 and its ligands is increased after irradiation. (a and b) Heatmap of chemokines and chemokine receptors through RNA sequencing in the liver, lung, and intestine of mice. (c) The mRNA levels of CXCR2 and its ligands CXCL1, CXCL2, CXCL3, and CXCL5 in the intestinal tissue of mice in the control group and the irradiation group (*n* = 4 per group). (d) Representative images of CXCR2 expression in the intestinal tissue of mice in the control group and the irradiation group by IHC staining at day 3. The scale bar is 50 *μ*m. ^∗^*p* < 0.05, ^∗∗^*p* < 0.01, ^∗∗∗^*p* < 0.001, and ^∗∗∗∗^*p* < 0.0001.

Compared with those in lung and liver tissues, the characteristic genes of neutrophils were most significantly upregulated in irradiated intestinal tissues, as shown in Figure 1(e). To explore the mechanism of irradiation-induced intestinal injury mediated by neutrophils, we focused on the expression of chemokines and chemokine receptors. By analysing and comparing various chemokines and their receptors through RNA sequencing, we found that CXCR2 and its ligands CXCL1, CXCL2, CXCL3, and CXCL5, as well as CCR3, FPR1, FPR2, and C5aR1, were significantly upregulated in irradiated intestinal tissue (see Figures 3(a) and 3(b)). We first verified CXCR2 and its ligands to clarify its relationship with irradiation-induced intestinal injury, which was also verified by PCR and immunohistochemistry.

First, PCR was used to detect the mRNA levels of CXCR2 and its ligands CXCL1, CXCL2, CXCL3, and CXCL5 in intestinal tissues of the two groups. The results showed that the mRNA levels of CXCR2 and its ligands were significantly increased in the irradiation group compared with the control group (see Figure 3(c)). Then, CXCR2 expression in the intestinal tissue of the two groups was further detected by immunohistochemistry, and the result was consistent with the change in mRNA levels in the two groups. The protein level of CXCR2 was significantly increased in the irradiation group (see Figure 3(d)).

### 3.4. CXCL-CXCR2 Promotes Neutrophil Chemotaxis and Intestinal Injury after Irradiation

**Figure 4:**
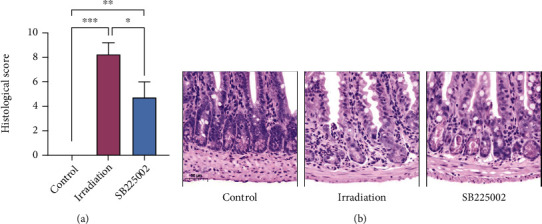
CXCL-CXCR2 promotes neutrophil chemotaxis and intestinal injury after irradiation. (a) Histological scores of intestinal tissues of mice in the control group, the irradiation group, and the irradiation+SB225002 group after H&E staining (*n* = 4 per group). (b) Representative images of the H&E staining of the intestine from the control group, the irradiation alone group, and the irradiation+SB225002 group at day 3. The scale bar is 50 *μ*m. ^∗^*p* < 0.05, ^∗∗^*p* < 0.01, ^∗∗∗^*p* < 0.001, and ^∗∗∗∗^*p* < 0.0001.

The results of RNA sequencing showed that the characteristic genes of neutrophils were most significantly upregulated in intestinal tissues compared with lung and liver tissues, suggesting that neutrophil infiltration may play a potential role in irradiation-induced intestinal injury. In addition, we noted that CXCR2 and its ligands CXCL1, CXCL2, CXCL3, and CXCL5 were significantly increased in irradiated intestinal tissue. To further investigate the effects of CXCR2 and its ligands on neutrophil infiltration and irradiation-induced intestinal injury, we injected an antagonist of CXCR2, SB225002 (2 mg/kg), into the mice intraperitoneally. The results of H&E staining showed that the infiltration of neutrophils in the intestinal tissue was significantly reduced and intestinal injury was alleviated after treatment with SB225002 compared with the irradiation alone group (see Figures 4(a) and 4(b)), indicating that the CXCL-CXCR2 signalling pathway is crucial for irradiation-induced intestinal injury by initiating neutrophil infiltration.

## 4. Discussion

The gastrointestinal system is sensitive to ionizing radiation, and radiation enteritis is common with radiotherapy of malignant tumours in the abdomen and pelvic cavity [1, 2]. Intestinal complications caused by irradiation, such as diarrhoea, malabsorption, bleeding, and perforation, seriously reduce the quality of life of patients and even affect clinical efficacy [4, 5]. Studies have shown that radiation enteritis is an inflammatory response in nature. Due to intestinal ionizing radiation and its complex healing mechanism, no universally accepted treatment method is available for radiation enteritis [32]. To explore potential intervention targets for radiation enteritis treatment, RNA sequencing was performed on liver, lung, and intestine tissues from nonirradiated mice and irradiated mice. We found that the expression of characteristic genes of neutrophils was significantly altered in intestinal tissue. We demonstrated that neutrophil infiltration is critical for irradiation-induced intestinal injury by clearing neutrophils with the anti-Ly6G antibody.

Chemokines are the main inflammatory factors mediating the directed chemotaxis of leukocytes in the process of inflammation [33–35]. According to the arrangement of amino terminal (N-terminal) cysteines, chemokines can be divided into four subfamilies—CXC, CC, XC, and CX3C—and the CXC subfamily mainly recruits neutrophils [36, 37]. Many studies have shown that chemokines and their receptors play an important role in the progression of inflammatory diseases and tumours [38–41]. By analysing and comparing various chemokines and chemokine receptors, we found that CXCR2 and its ligands CXCL1, CXCL2, CXCL3, and CXCL5 in intestinal tissues were all significantly upregulated, which was also verified by PCR and immunohistochemistry techniques. The results showed that CXCR2 and its ligands were significantly increased in the irradiation group, which was consistent with the results of RNA sequencing.

CXCR2 is a G-protein-coupled receptor that can recruit neutrophils to the site of injury and inflammation by binding to its ligands [42]. Compared with that in lung and liver tissues, we found that irradiation could significantly increase the expression of CXCR2 and its ligands CXCL1, CXCL2, CXCL3, and CXCL5 in intestinal tissues. Therefore, we speculated that CXCR2 and its ligands may be related to irradiation-induced intestinal injury and neutrophil infiltration. To test our hypothesis, we used the CXCR2 antagonist SB225002 to block the CXCR2 signalling pathway and inhibit the chemotaxis of neutrophils via the CXCL-CXCR2 axis. The results showed that SB225002 reduced the aggregation and infiltration of neutrophils to the inflammatory site and alleviated intestinal injury. Our study provides a new experimental basis and theoretical basis for the clinical treatment of radiation enteritis and may become a new therapeutic target.

## 5. Conclusions

In summary, our study revealed that the characteristic genes of neutrophils were significantly upregulated in irradiated intestinal tissue. Elimination of neutrophils with anti-Ly6G antibody relieved irradiation-induced intestinal injury. In addition, CXCR2 and its ligands were significantly increased in irradiated intestinal tissue. The CXCR2 antagonist SB225002 reduced the infiltration of neutrophils and alleviated intestinal injury. Targeting CXCR2 and its ligands may provide a novel therapeutic target for radiation enteritis.

## Data Availability

The data used to support the findings of this study are included within the article. Requests for data, 6 months after the publication of this article, will be considered by the first author.
